# β1 integrin signaling in asymmetric migration of keratinocytes under mechanical stretch in a co-cultured wound repair model

**DOI:** 10.1186/s12938-016-0263-1

**Published:** 2016-12-28

**Authors:** Dongyuan Lü, Zhan Li, Yuxin Gao, Chunhua Luo, Fan Zhang, Lu Zheng, Jiawen Wang, Shujin Sun, Mian Long

**Affiliations:** 1 Center of Biomechanics and Bioengineering, Institute of Mechanics, Chinese Academy of Sciences, Beijing, 100190 China; 2 Key Laboratory of Microgravity (National Microgravity Laboratory), Institute of Mechanics, Chinese Academy of Sciences, Beijing, 100190 China; 3 Beijing Key Laboratory of Engineered Construction and Mechanobiology, Institute of Mechanics, Chinese Academy of Sciences, Beijing, 100190 China

**Keywords:** Keratinocyte, Mechanical stretch, Fibroblast, β1 integrin, Mechanotransduction

## Abstract

**Background:**

Keratinocyte (KC) migration in re-epithelization is crucial in repairing injured skin. But the mechanisms of how mechanical stimuli regulate the migration of keratinocytes have been poorly understood.

**Methods:**

Human immortalized keratinocyte HaCaT cells were co-cultured with skin fibroblasts on PDMS membranes and transferred to the static stretch device developed in-house for additional 6 day culture under mechanical stretch to mimic surface tension in skin. To detect the expression of proteins on different position at different time points and the effect of β1 integrin mechanotransduction on HaCaT migration, Immunofluorescence, Reverse transcription-polymerase chain reaction, Flow cytometry, Western blotting assays were applied.

**Results:**

Mechanical receptor of β1 integrin that recognizes its ligand of collagen I was found to be strongly associated with migration of HaCaT cells since the knockdown of β1 integrin via RNA silence eliminated the key protein expression dynamically. Here the expression of vinculin was lower but that of Cdc42 was higher for the cells at outward edge than those at inward edge, respectively, supporting that the migration capability of keratinocytes is inversely correlated with the formation of focal adhesion complexes but positively related to the lamellipodia formation. This asymmetric expression feature was further confirmed by high or low expression of PI3K for outward- or inward-migrating cells. And ERK1/2 phosphorylation was up-regulated by mechanical stretch.

**Conclusion:**

We reported here, a novel mechanotransduction signaling pathways were β1 integrin-dependent pattern of keratinocytes migration under static stretch in an in vitro co-culture model. These results provided an insight into underlying molecular mechanisms of keratinocyte migration under mechanical stimuli.

## Background

Wound healing is an intricate process in which the skin repairs itself with a series of sequential cellular and biochemical events after injury [1]. It is usually divided into three or four sequential yet overlapping phases, including hemostasis, inflammation, granulation tissue formation and re-epithelialization, matrix formation and remodeling [2, 3]. All of these phases are highly coordinated physiological processes and require dynamic, coordinated intercommunication among different type cells in specific tissue regions. Keratinocytes are recognized to regulate evidently wound repairing through cell migration, proliferation and differentiation, especially in the crucial step of re-epithelialization [1, 4]. Re-epithelization is a key procedure during wound repairing where keratinocytes migrate asymmetrically to cover the wound bed prior to cell proliferation in a few hours after wounding [5]. Currently, keratinocyte migration dynamics acts as an excellent model for elucidating the wound healing both in vivo and in vitro.

It has been well known that keratinocyte migration dynamics is highly manipulated by their host microenvironment [1, 6]. On a hand, biochemical signaling is crucial to cell migration, including the intercommunication with other dermal cells, extracellular matrix (ECM), or growth factors and cytokines produced by fibroblasts [1]. Distinct constituents of ECM have different effects on keratinocyte migration velocity and motility [7–9]. Specifically, type I collagen, as one of main ECM components in wound site, plays a crucial role in modulating keratinocyte migration [2]. On the other hand, mechanical signaling is also an important factor in wound repairing because the configuration and function of regenerative tissue depends on skin contraction. For example, the contractile activity can be enhanced between keratinocyte and fibroblast interactions under mechanical tension [10]. Mechanical forces derived from tissue development and remodeling regulate the synthesis of various ECM components and speed the wound healing progress [11, 12]. Keratinocyte migration mediated by collagen I involves in the binding of cell surface adhesive receptors to matrix proteins in which mechanical forces play a crucial role in modulating the de novo synthesis of collagens. Clinically, although the topical suction pressure therapy, vacuum-assisted closure (VAC), has been known as an effective, widely-applied technique to promote various chronic wounds healing [13, 14], it is still unclear why the mechanical forces derived by suction pressure is beneficial in the VAC therapy at cellular as well as molecular levels [15]. Previously, we found that HaCaT tends to migrate asymmetrically under mechanical stretch in the presence of fibroblast co-culture, which is mainly mediated by EGF in a paracrine manner [16]. However, the underlying mechanisms in intracellular signaling remain unknown.

To date, cell mechanotransduction is known to be a well-defined process to translate extracellular mechanical signals into intracellular biochemical events. For example, a mechanical receptor of β1 integrin expressed on keratinocyte surface (e.g., α2β1, and α3β1) is able to sense the mechanical signals via binding to the surrounding collagen I [17, 18]. There is growing evidence to support that β1 integrin is a key adhesive molecule in de novo focal contact formation, keratinocyte migration, and re-epithelization of wound tissue [19, 20]. Migratory capacity of β1-deficient keratinocytes is strongly impaired in vitro and epithelial migration is dramatically reduced in wound healing in β1-integrin null mice [20]. Although β1 integrin is a well-known mechanosensor for various types of cells, little is known about its roles in keratinocyte mechanotransduction mechanisms as well as the underlying transduction pathways [21, 22]. Not only the so-called “outside-in” signaling induces the formation of focal adhesion complex (FAC) and the remodeling of actin polymerization, but it also activates the downstream phosphorylation cascade of intracellular stretch-sensitive proteins and the expression of mechanically-sensing genes to regulate a variety of cellular functions, such as cell migration [23]. For example, integrins regulate various protein kinases (e.g., tyrosine kinase, phosphatase, and mitogenesis-associated protein kinase or MAPK) in cell proliferation and other processes [21, 24]. As a key signaling molecule in MAPK pathway, extracellular-signal-regulated kinase (ERK1/2) activation is specifically required in epithelial cell migration where ERK1/2 pathway coordinates the dynamics of wound healing and the inhibition of ERK1/2 delays the process of wound healing [25]. Moreover, ERK1/2 also plays a crucial role in mediating cellular responses to mechanical stretch. Combined with the phosphatidylinositol-3-OH kinases (PI3Ks) that serve as the mediators of integrin-induced cytoskeletal remodeling and cell migration [26, 27], the other small GTPases, such as Cdc42, regulate actin polymerization in a collagenous matrix and modulate the motility and invasion of epithelial cells in a PI3K-dependent pathway [28]. Thus, it is important to elucidate the underlying pathways of β1 integrin-induced keratinocytes migration under mechanical stimuli.

Together, the challenging issues for mechanotransduction mechanisms of keratinocytes migration in cutaneous wounds mainly rely on: Whether does mechanical stretch modulate dynamically the expression of key signaling proteins and how do the cells sense the mechanical signals? Whether do the other signaling factors affect the migration of keratinocytes and what are the potential mechanotransduction mechanisms? Here we developed an in vitro static stretch approach to quantify the mechanically-induced proteins expression of human keratinocytes on substrate coated by collagen I and in the presence of human fibroblasts. Mechanotransduction mechanisms of β1 integrin-mediated signaling pathway were determined. Our results provided the insight into the mechanotransduction pathways in manipulating keratinocyte migration under static stretch, which implies potential application in clinical treatment of wound repairing.

## Methods

### Cell lines and reagents

**Fig. 1:**
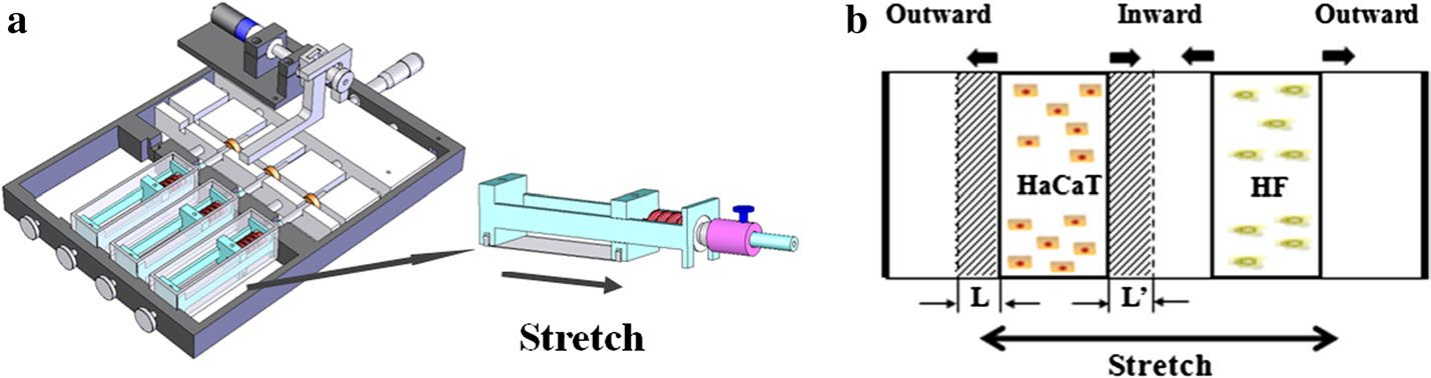
Mechanical stimuli used to examine signaling proteins in HaCaT cells migration. **a** Image of an in-house developed static stretch device by applying mechanical stimuli via a stretchable silicone membrane to the cells. **b** Schematic of cell migration under tensile stress on silicone membrane at a typical 20% strain. HaCaT and HF cells were seeded in two separated regions, and the migration distance L (away from HF cells) or L’ (towards HF cells) and the migration leading edge of HaCaT cells were illustrated

Human immortalized keratinocyte HaCaT cell line CRL2309 and human skin fibroblast (HF) cell line CRL2088 were obtained from American Type Culture Collection (ATCC, Rockefeller, USA). HaCaT cells were grown in RPMI 1640 medium (Hyclone, Utah, USA) with 10% fetal bovine serum (FBS, Gibco, Grand Island, USA) and 1% penicillin/streptomycin (Hyclone, Utah, USA). Fibroblasts were cultured in Dulbecco’s Modified Eagle’s medium (DMEM, 1 g/liter glucose) with 10% FBS and 1% antibiotics. Cells were dissociated using 0.05% trypsin and 0.02% EDTA in phosphate-buffered saline (PBS, pH 7.4) when they are approximately 85–90% confluent, and moved to the static stretch device developed in-house for additional 6 day culture under mechanical stretch (Fig. 1a) [16]. Cells were detected at different time points for functional measurements of mechanotransduction pathways.

Mouse-anti-human anti-β1 integrin monoclonal antibodies (mAbs) for flow cytometry and western blotting (WB), anti-β-actin mAb for WB, anti-Cdc42, anti-PI3K, and anti-phosphorylated (*p*-) ERK1/2 mAbs as well as goat-anti-human polyclonal antibody against vinculin for immunofluorescence (IF) staining, were obtained from Santa Cruz Biotechnology (Dallas, Texas, USA). Alexa Fluor-conjugated secondary mAbs (Sigma-Aldrich, Missouri, Saint Louis, USA) for IF staining and HRP-conjugated secondary mAbs (Boster, Wuhan, Hubei, China) for WB analysis were obtained. Coverslips and paraformaldehyde were purchased from Fisher Scientific (Somerville, Massachusetts, USA). Hygromycin B was from Roche (Baden-Wuerttemberg, Mannheim, Germany). Acid-soluble bovine achilles tendon-derived collagen I (cell matrix type I-A), bovine serum albumin (BSA), and sodium dodecyl sulfate (SDS) were obtained from Sigma-Aldrich.

### Cells migration

To detect the dynamics of protein expression at the migration leading edge of HaCaT cells inwardly (between two cell zones) or outwardly (away from two cell zones) directed to co-cultured fibroblasts, cells (keratinocytes or fibroblasts) seeding and migration were performed as described previously [16]. To exclude the possible impacts of cell proliferation on migration dynamics of HaCaT cells, both types of cells were pre-incubated with a conventional cell proliferation inhibitor (mitomycin C at 10 μg/ml for 2 h) prior to seeding them onto silicone membrane and exerting mechanical stretch [16]. Mitomycin C-treated HaCaT or HF cells grown in a flask were transferred onto silicone membrane pre-coated by collagen I in 0.15 mg/ml at 37 °C for 2 h or treated by oxygenized plasma as a control. To mimic the distributions of the two types of cells in separated regions in wound repair, HF cells were put only on one side of HaCaT cells. Nontoxic stainless steel frames were used retained the cells inside the seeding zone. The membrane was then mounted to the stretch device and experienced a steady stretch of 20% strain for 6 days to the HaCaT and HF co-culture (Fig. 1b). HaCaT and HF cells were seeded at respective density of 5 × 10^5^ cells/0.8 cm^2^ and 1 × 10^5^ cells/0.8 cm^2^. Optical images of cell migration leading edge were monitored using a CCD-camera at the particular time point.

### IF staining

To quantify the time course of the expressions of vinculin, Cdc42, PI3K and phosphorylated ERK1/2 in HaCaT cells under co-cultured and mechanical stretch, HaCaT cells adhering on the silicone membrane at different time points were fixed with 4% paraformaldehyde in PBS for 15 min, permeated with 0.2% Triton X-100 for 4 min, and blocked by 1% BSA for 30 min at room temperature. The cells were then incubated with relevant primary antibodies, respectively, for 1 h at 37 °C or overnight at 4 °C (1:100 dilution in 1% BSA). After washing, rhodamine-conjugated second antibody was added in for additional 45 min incubation at room temperature. A coverslip was mounted onto silicone membrane in FluoPrep mounting medium (Dako, Trappes, France) and the cells locating at the leading edge of migration zone were visualized by a Tcs sp5 Leica confocal laser microscope (Leica, Cambridge, UK). Fluorescent images were captured for ~20 cells in one frame and totally three frames in each case. Image analysis was done using by ImageJ 1.41 software (National Institutes of Health, Bethesda, USA) to calculate the fluorescent intensity of the stained individual cells by setting a threshold. Normalized mean fluorescence intensity (FI) was used to indicate the relative fluorescent intensity of detected proteins.

### RNA interference of β1 integrin in HaCaT

p*Silencer* hygro plasmid (Ambion, Austin, TX, USA) was used for DNA vector-based RNA interference. The β1 integrin RNAi plasmid was structured based on p*Silencer* hygro plasmid (Plasmids as the gift from Dr. Xiangdong Luo, Third Military Medical University). RNA interference experiments were carried out using commercial reagent upon the manufacturer’s instructions. Briefly, the RNAi plasmids were transfected into HaCaT cells using Lipofectamine™ 2000 reagent (Invitrogen, Carlsbad, USA) in 1–2 μg of expression plasmid in a 6 well plate with serum-free medium. After 6 h of transfection, the medium was replaced by serum-containing medium and incubated for 48 h. Collected cells were then grown in the medium of RPMI 1640 containing hygromycin B (80 μg/ml) to enrich the successfully-transfected cells and to select the cell subpopulation expressing stably the target siRNA. Stably-silenced β1 integrin HaCaT cell population was then cultured in standard condition (37 °C with 5% CO_2_) with hygromycin B (80 μg/ml) supplied in medium. Culture medium was exchanged each 3 or 4 days, and the knockdown efficiency of β1 integrin expression after 21-day cell culture was confirmed by WB, RT-PCR, and flow cytometry tests. Negative and positive controls were designed to exclude the nonspecific effects.

### WB assay

To detect the knockdown efficiency of β1 integrin in HaCaT, cells were harvested and lysed with ice-cold modified RIPA buffer (50 mM Tris–Cl at pH 7.4, 1% NP40, 150 mM NaCl, 1 mM EDTA, 1 mM PMSF, 1 mM Na_3_VO_4_, 1 mM phosphatase inhibitors, and 5 mg/ml each of aprotinin, leupeptin, and pepstatin). After being sonicated for 30 s, lysates were maintained on ice for 30 min, boiled for 5 min and then clarified by centrifugation for 10 min at 12,000*g*. Collected supernatant was used for WB analysis and protein concentrations were determined using a BCA protein assay kit (Pierce, Rockford, USA) with BSA as a standard. Briefly, same amounts of proteins were separated by electrophoresis on SDS–polyacrylamide gel and electroblotted onto nitrocellulose (NC) filters. Both the NC membranes and the blots were blocked with TBS-T (10 mM Tris–Cl at pH 8.0, 150 mM NaCl, 0.05% Tween-20) containing 5% nonfat dried milk for >1 h at room temperature. Anti-β1 integrin and anti-β-actin mAbs were added in and incubated overnight at 4 °C and washed three times in TBS-T, respectively. Protein blots were then incubated with a HRP-conjugated secondary mAb for 1 h at room temperature and visualized on X-ray films using enhanced chemiluminescence (Amersham Pharmacia Biotech, Piscataway, USA). β1 integrin in wild-type HaCaT was detected as the positive control.

### RT-PCR

RT-PCR was performed to screen the HaCaT cell clones with stable knockdown of β1 integrin. Total RNA was extracted using RNAiso Plus and subjected to reverse transcription into cDNA using PrimeScript 1st Strand cDNA synthesis Kit (TAKARA, Dalian, China). Briefly, 1 μg of RNA was reverse transcribed using oligo (dT) as primer in a total volume of 25 μl. 5 μl of cDNA solution was used to amplify specific transcripts by PCR. For semi-quantitative PCR of β1 integrin, amplification of both β1 integrin (Accession: BC020057.1) and β-actin genes (Accession: X00351.1) were conducted in the same tube. Regular PCR was done using *Taq* polymerase on the following primers 5′-GGA AAA CGG CAA ATT GTC AG-3′ and 5′-TTG GGG TTG CAC TCA CAC AC-3′ for amplification of β1 integrin (600 bp), and 5′-CGT GGA CAT CCG CAA AGA C-3′ and 5′-CTG CTG TCA CCT TCA CCG TTC-3′ for amplification of β-actin (441 bp) for 35 cycles (94 °C for 5 min, 30 cycles at 94 °C for 30 s, 55 °C for 30 s, 72 °C for 30 s) and finally extension at 72 °C for 7 min. The products were then visualized by 1.5% agarose gel electrophoresis and subsequent ethidium bromide staining.

### Flow cytometry

Monolayer HaCaT cells were harvested and neutralized by adding medium containing FBS. After being washed twice in PBS, the suspension of HaCaT cells was incubated with anti-β1 integrin mAb in 1 μg per 1 × 10^6^ cells for 1 h on ice, and subsequently labeled with fluorescein-conjugated secondary antibody for 45 min on ice (1:500 dilution). After washing three times in PBS, collected cells were tested using a FACSCalibur machine (Becton–Dickinson, San Jose, USA) and the data were analyzed using FACSDiva software.

### Data analysis

All data were collected from at least triplet measurements and presented as mean ± standard error (SE). Analysis of variance (ANOVA) was used to compare the differences among various groups, and Student *t* test was employed to compare the difference between two groups. *P* value indicates the level of statistical significance of differences in the normalized distance or fraction. Tests that produce *P* < 0.01 were considered to be significant.

## Results

### β1 integrin mediates HaCaT migration

**Fig. 2:**
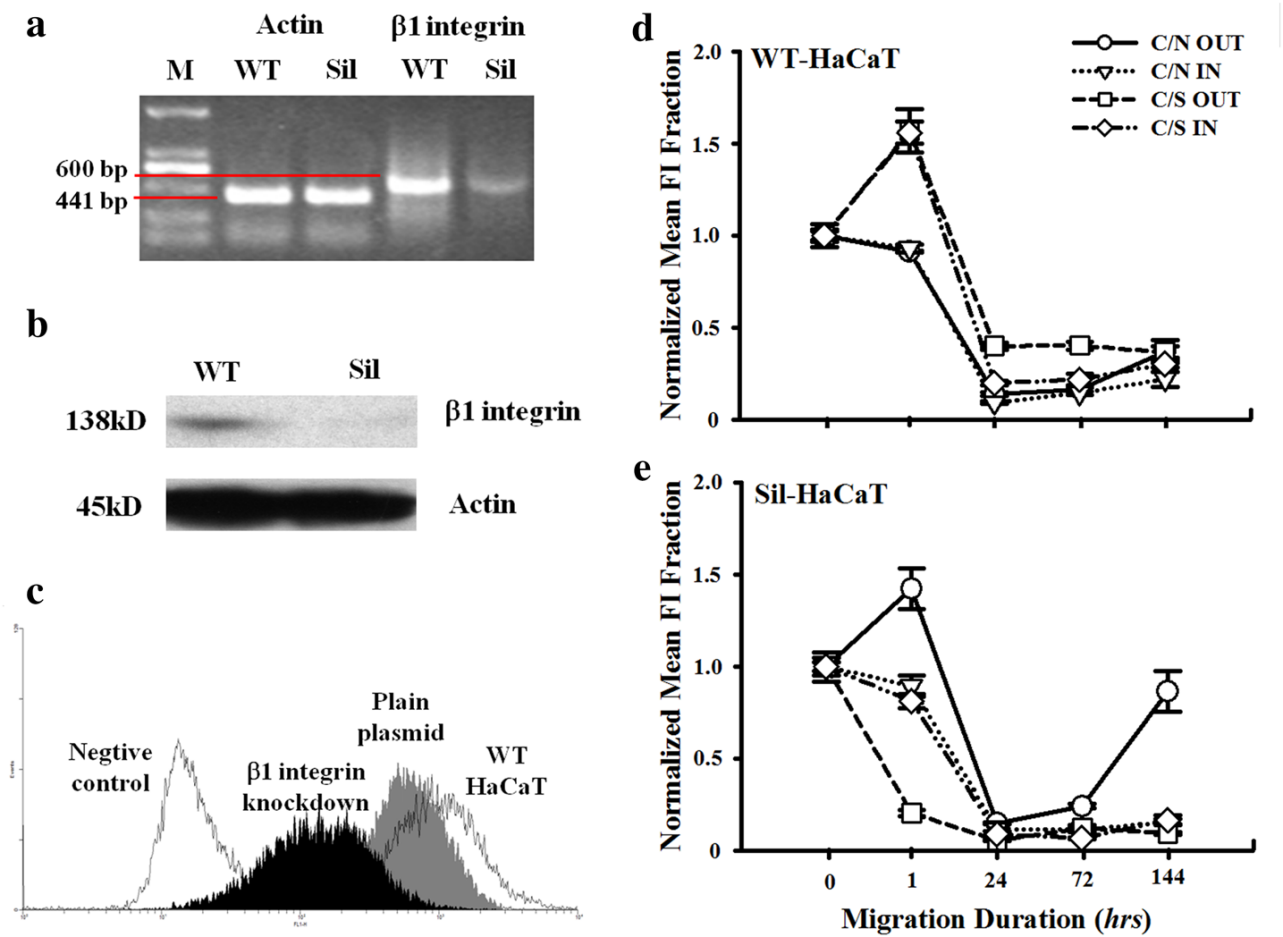
Alteration of β1 integrin expression on WT- and β1 integrin knockdown- HaCaT cell migration under coculture and mechanical stretch. **a** RT-PCR analysis of β1 integrin in WT- and silenced- (Sil-) HaCaT cells. *Red lines* indicated the molecular weight of the target fragments. **b** WB analysis of β1 integrin in WT- and Sil-HaCaT cells. **c** Comparison of β1 integrin expression between WT- (*open bar*) and Sil- (*solid bar*) HaCaT cells. WT-cells transfected via plain plasmid (*grey bar*) was used as control. **d**, **e** Time courses of β1 integrin expression in WT- (**d**) or Sil- (**e**) HaCaT cells under cocultured with fibroblasts and mechanical stretch. Data were presented as the mean ± standard error (*SE*) of normalized fluorescence intensity (*FI*) fold of totally >9 cells at the leading edge

The cascade of cell migration includes the cell adhesion to ECM, the formation of FACs, and the remodeling of actin cytoskeleton, in which β1 integrin is thought to be an important mechanical receptor in retaining the directed trajectory of keratinocyte migration [29]. To identify the impact of β1 integrin on keratinocyte migration, we knocked down its expression by transfecting a β1 integrin-p*Silencer* plasmid into HaCaT cells (named as Sil-HaCaT) when a mock plasmid served as a control. The efficiency of β1 integrin knockdown in stably-transfected HaCaT cells was tested using semi-quantitative PCR analysis at RNA level (Fig. 2a), WB test (Fig. 2b) and flow cytometry analysis (Fig. 2c) at protein level. These results confirmed that the expression of β1 integrin was high in wild-type (WT) HaCaT but quite low in Sil-HaCaT cells. To further understand the role of β1 integrin in the dynamics of HaCaT migration, we compared the time course of β1 integrin expression of WT- (Fig. 2d) and Sli- (Fig. 2e) HaCaT cells at both the leading edges under co-cultured and mechanical stretch. On one hand, the expression under stretch was enhanced in WT-cells at both the outward (*squares*) and inward (*diamonds*) leading edges at 1 h followed by a reduction down to the baseline level for up to 144 h, suggesting that the up-regulation of β1 integrin expression under mechanical stretch exhibited a rapid transition phase and no differences were observed between outward and inward migration of HaCaT cells. By contrast, the expression under non-stretch monotonically decreased with time and again no differences were observed between outward (*cycles*) and inward (*triangles*) migration (Fig. 2d). This time-lapsed declination of MFI per unit area is simply because the spreading area of those cells at leading edge keep increasing with time when β1 integrin expression tends to be stable at sufficiently long time. On the other hand, the stretch-induced rapid up-regulation of β1 integrin expression found for WT-cells was no longer present in Sil-cells, supporting that β1 integrin is the mechanical receptor to sense the static stretch. Here no significant difference in normalized MFI was observed for C/N OUT case between 0 and 1 h even though mean value at 1 h was slightly higher (1.00 ± 0.07 vs. 1.42 ± 0.16, *P* = 0.077), which is likely attributed to very limited spreading of HaCaT cells within 1 h and quite low expression of β1 integrin in Sil-HaCaT cells. Meanwhile, slight differences between outward (*squares* or *cycles*) and inward (*diamonds* or *triangles*) leading edges were found under stretch or non-stretch (Fig. 2e), presumably attributed to the mechanical and/or biochemical sensing of other surface receptors rather than β1 integrin since the absolute β1 integrin expression was quite low in Sil-HaCaT cells. Together, these results indicated β1 integrin serves as a mechanical receptor to translate the extracellular mechanical signals into intracellular biochemical events in a rapid response even though no visible differences of β1 integrin expression were found between the two leading edges, suggesting that β1 integrin is the key regulator of mechanical signals to alter the magnitude and pattern of HaCaT migration on collagen I-coated substrate.

### Vinculin plays pivotal roles in HaCaT migration

**Fig. 3:**
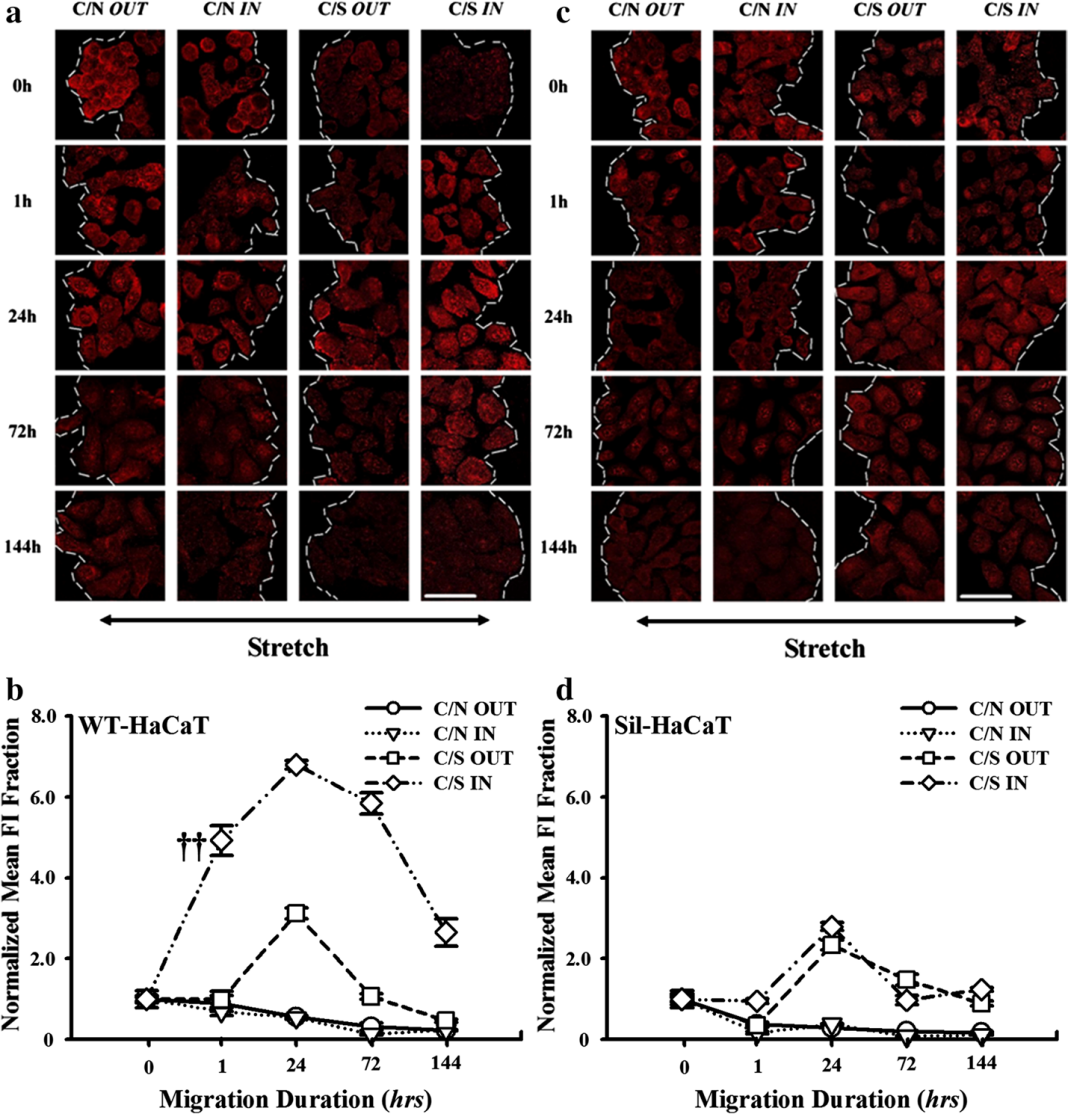
Expression of vinculin in WT- and Sil-HaCaT cells in four migration patterns at different time points. **a**, **b** IF images and time courses of vinculin expression in WT-HaCaT cells (**a**). Data were presented as the mean ± SE of normalized *FI* of totally >9 cells at the leading edge. ^††^The level of statistical significance of difference in normalized mean *FI* at *t* = 1 h between *C/S IN* and *C/S OUT* patterns in WT-HaCaT cells (**b**). **c**, **d** IF images and time courses of vinculin expression in Sil-HaCaT cells (**c**). Data were presented as the mean ± SE of normalized *FI* of totally >9 cells at the leading edge (**d**). *Scale bar* 50 μm

Outside-in signaling via β1 integrin-collagen I interactions activates the formation of FACs that possesses the mechanical resistance to the applied stretch. Since vinculin-associated focal contacts are thought to be in migratory phenotype and β1 integrin is able to anchor to the FACs via its cytoplasmic tail [30–33], we next tested the impact of vinculin expression on HaCaT migration. For co-cultured WT-HaCaT cells, time course of vinculin expression under stretch exhibited an ascending phase when *t* < 24 h followed by a descending phase and presented a declined phase without stretch. Interestingly, vinculin expression at the outward leading edge (*squares*) was dramatically lower than that at the inward edge (*diamonds*) under mechanical stretch (e.g., *P* = 0.0014 at *t* = 1 h), indicating that HaCaT cells prefer to migrate to the outward end due to the down-regulation of vinculin as well as FACs. By contrast, no differences were found at the inward (*triangles*) and outward (*cycles*) edges under non-stretch (e.g., *P* = 0.829 at *t* = 1 h), all of which were significantly lower than those under stretch, respectively (Fig. 3a, b). These results indicated that the up-regulation of vinculin expression of co-cultured HaCaT cells is mechanically-dependent and that the asymmetric presentation of vinculin molecules is positively correlated with the asymmetric migration under stretch. Conversely, no differences of vinculin expression were observed at the inward and outward edges when the Sil-HaCaT cells were co-cultured with HF cells under stretch (Fig. 3c, d), suggesting that the function of vinculin is well correlated with that of β1 integrin.

### Cdc42 is important in HaCaT migration

**Fig. 4:**
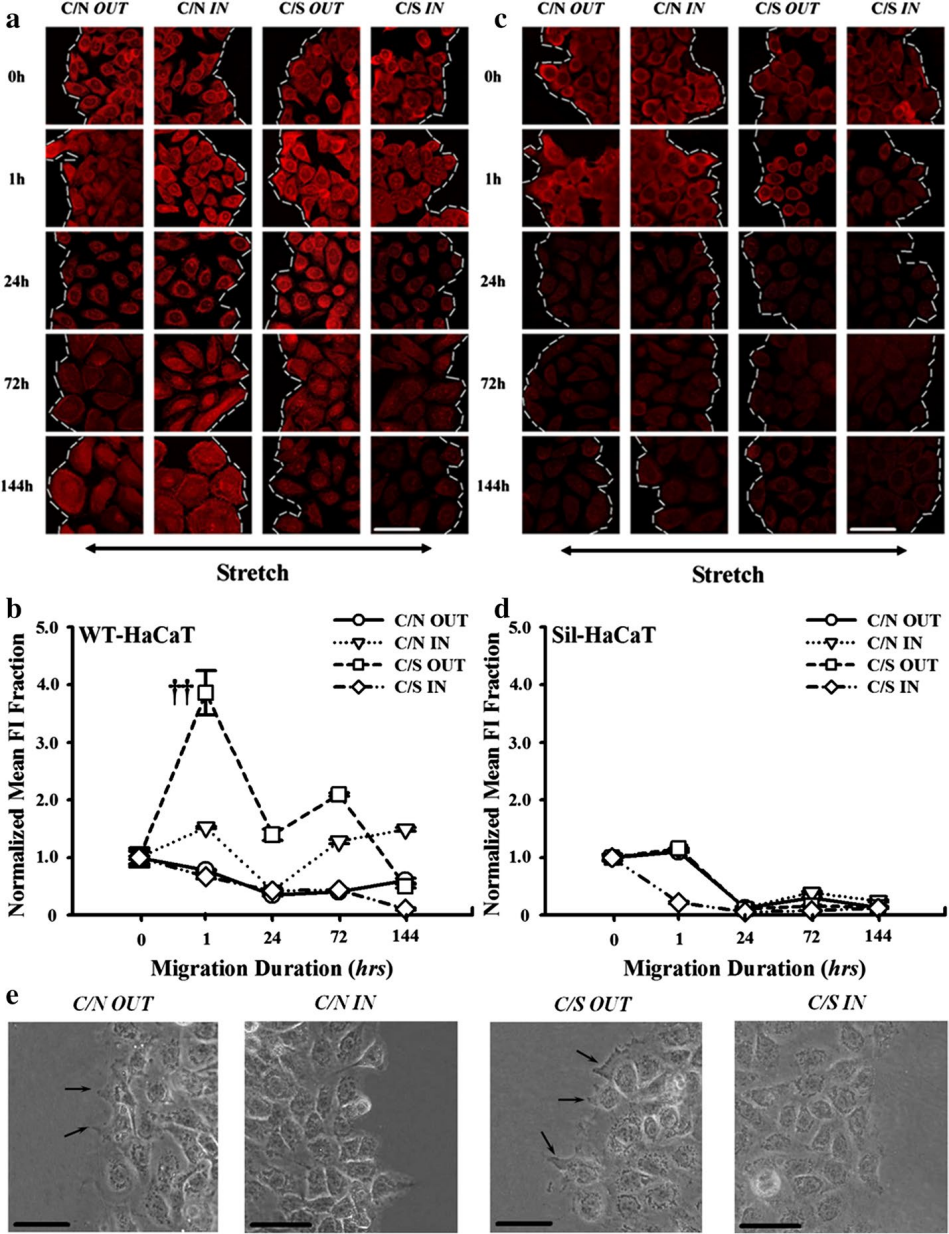
Expression of Cdc42 in WT- and Sil-HaCaT cells in four migration patterns at different time points. **a**, **b** IF images and time courses of Cdc42 expression in WT-HaCaT cells (**a**). Data were presented as the mean ± SE of normalized *FI* of totally >9 cells at the leading edge. ^††^The level of statistical significance of difference in normalized mean *FI* at *t* = 1 h between *C/S IN* and *C/S OUT* patterns in WT-HaCaT cells (**b**). **c**, **d** IF images and time courses of Cdc42 expression in Sil-HaCaT cells (**c**). Data were presented as the mean ± SE of normalized *FI* of totally >9 cells at the leading edge (**d**). **e** Optical images of pseudopodium formation in WT-HaCaT cells. *Arrows* indicate the pseudopodium at day 2. *Scale bar* 50 μm

We further tested if Rho family GTPase, particularly Cdc42, is involved in β1 integrin-mediated HaCaT migration since they are key regulators of cell motility, contractility, and migration through the linkage between integrins and cytoskeletal proteins [34, 35]. Similarly, time course of Cdc42 expression of co-cultured WT-HaCaT cells under stretch exhibited a rapid increase up to *t* = 1 h followed by a descending phase. And the expression at the outward edge (*squares*) was higher than that at the inward edge (*diamonds*) (e.g., *P* = 0.000045 at *t* = 1 h), indicating that HaCaT cells prefer to form the lamellipodia at the outward end due to the up-regulation of Cdc42 molecules [36]. By contrast, no significant differences were found at the inward (*triangles*) and outward (*cycles*) edges without stretch (e.g., *P* = 0.141 at *t* = 1 h) (Fig. 4a, b). These results indicated that the mechanically-dependent up-regulation of Cdc42 expression at the outward edge of co-cultured HaCaT cells, which is favorable to cell spreading, is positively correlated with the asymmetric migration under stretch. Conversely, no differences of Cdc42 expression were observed at the inward and outward edges when the Sil-HaCaT cells were co-cultured with HF cells under stretch (Fig. 4c, d), suggesting that the function of Cdc42 is well correlated with that of β1 integrin. It was also found that pseudopodium is more readily visible in C/S outward edge than that in C/N, but hard to be visualized in inward edge regardless of C/S or C/N (Fig. 4e).

### PI3K and ERK1/2 signaling participates in β1 integrin-mediated HaCaT migration

**Fig. 5:**
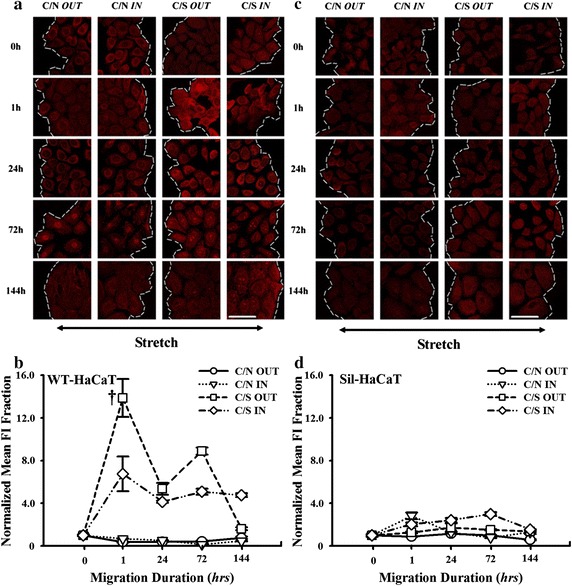
Expression of PI3K in WT- and Sil-HaCaT cells migration in four migration patterns at different time points. (**a**, **b**) IF images and time courses of PI3K expression in WT-HaCaT cells (**a**). Data were presented as the mean ± SE of normalized *FI* of totally >9 cells at the leading edge. ^†^The level of statistical significance of difference in normalized mean *FI* at *t* = 1 h between *C/S IN* and *C/S OUT* patterns in WT-HaCaT cells (**b**). (**c**, **d**) IF images and time courses of PI3K expression in Sil-HaCaT cells (**c**). Data were presented as the mean ± SE of normalized *FI* of totally >9 cells at the leading edge (**d**). *Scale bar* 50 μm

We also tested the downstream signaling pathways that are associated with cell migration. For example, PI3Ks are involved in many cellular functions such as cell growth, motility, survival, and intracellular trafficking [37]. In the current work, it was found that PI3K expression for co-cultured WT-HaCaT cells was significantly enhanced at 1 h followed by a decrease or fluctuation, whereas it almost retained the same level without stretch. Importantly, the stretch-induced expression at the outward edge (*squares*) is dramatically higher than that at the inward leading edge (*diamonds*) (*P* = 0.0429), indicating that HaCaT cells prefer to migrate to the outward end due to the up-regulation of PI3K. By contrast, no differences were found at the inward (*triangles*) and outward (*cycles*) edges under non-stretch (*P* = 0.916), all of which are significantly lower than those under stretch, respectively (Fig. 5a, b). These results indicated that the up-regulation of PI3K expression is mechanically-dependent and that the asymmetric presentation of PI3K molecules is positively correlated with the asymmetric migration under stretch. Conversely, no differences were observed between outward and inward edges for Sil-HaCaT cells under stretch or non-stretch (Fig. 5c, d), suggesting that the function of PI3K is well correlated with the presence of β1 integrin.

**Fig. 6:**
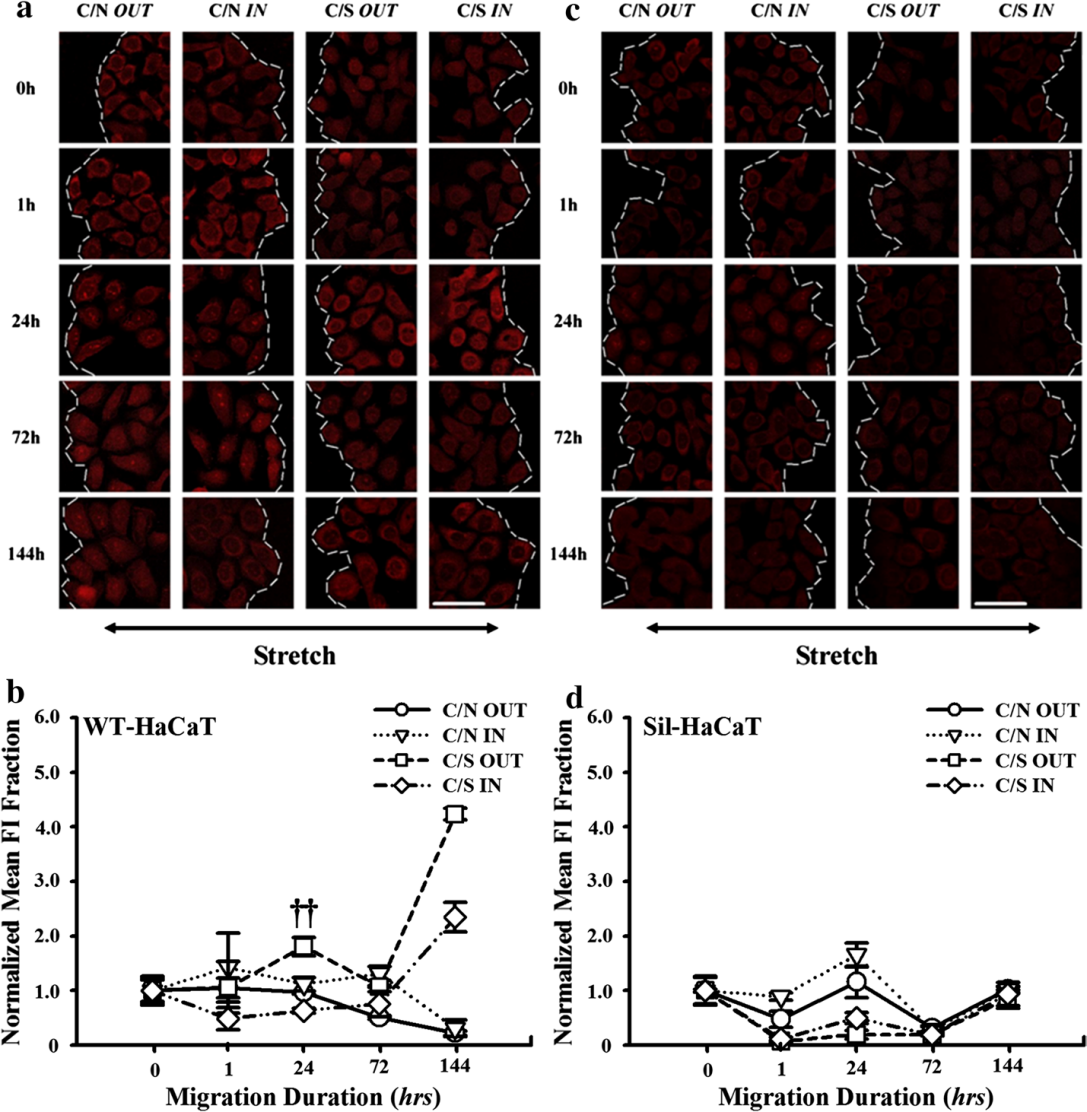
Expression of phosphorylated ERK1/2 in WT- and Sil-HaCaT cells in four migration patterns at different time points. **a**, **b** IF images and time courses of phosphorylated ERK1/2 expression in WT-HaCaT cells (**a**). Data were presented as the mean ± SE of normalized *FI* of totally >9 cells at the leading edge. ^††^The level of statistical significance of difference in normalized mean *FI* at *t* = 24 between *C/S IN* and *C/S OUT* patterns in WT-HaCaT cells (**b**). (**c**, **d**) IF images and time courses of phosphorylated ERK1/2 expression in Sil-HaCaT cells (**c**). Data were presented as the mean ± SE of normalized *FI* of totally >9 cells at the leading edge (**d**). *Scale bar* 50 μm

Activation of ERK1/2 pathway via β1 integrin, a well-known signaling, was also tested here for co-cultured WT- and Sil-HaCaT cells with or without mechanical stretch. Phosphorylated ERK1/2 expression in WT-HaCaT of C/S condition was altered obviously compared to that of C/N, especially at 24 h. Importantly, the stretch-induced expression at the outward edge (*squares*) is higher than that at the inward leading edge (*diamonds*) (*P* = 0.002), indicating that HaCaT cells prefer to migrate to the outward end due to activating ERK1/2. By contrast, no differences were found at the inward (*triangles*) and outward (*cycles*) edges under non-stretch (*P* = 0.307), all of which are lower than those under stretch, respectively (Fig. 6a, b). These results indicated that the up-regulation of phosphorylated ERK1/2 expression is mechanically-dependent and that the asymmetric presentation of phosphorylated ERK1/2 is positively correlated with the asymmetric migration under stretch. Conversely, no differences were observed between outward and inward edges for Sil-HaCaT cells under stretch or non-stretch (Fig. 6c, d). These results suggested that the capability of ERK1/2 mechanosensing and phosphorylation is reduced dramatically after β1 integrin knockdown. The activation of ERK1/2 is well correlated with the presence of β1 integrin.

## Discussion

The goal of the current work is to understand the dynamics of molecular mechanisms in keratinocyte migration under mechanical stimuli and co-cultured fibroblasts in wound repair model. Upon an in-house developed device of mechanical stretch, the capacity of HaCaT migration is asymmetric and prefers to migrate outwards, which is mainly governed by EGF factor mainly secreted by HF cells under mechanical stretch [16]. In the current work, those signaling proteins in asymmetric migration of HaCaT cells onto collagen I-coated substrate was directly visualized under mechanical stretch and in the presence of co-cultured HF cells. Cell signaling via collagen I-β1 integrin-vinculin pathway plays a pivotal role in mediating the keratinocytes migration. Downstream transduction of Cdc42, PI3K, and ERK1/2 phosphorylation was positively correlated with the specified migration of keratinocytes, which are all β1 integrin-dependent.

In fact, wound healing requires the cooperative interactions between different types of tissue and cell. The asymmetric migration of keratinocytes is the result of multiple interactions, including ECM, fibroblasts, and mechanical stretch, to affect the migration dynamics of skin keratinocytes. Since asymmetric migration might be an important behavior of cells to build tissues and organs (in epithelial cells to shape epithelial structures), here we revealed the interplay of specific molecular signaling pathways (β1 integrin, vinculin, Cdc42, PI3K, and ERK1/2) involved in asymmetric migration, this work is helpful to better understand how cells work for autonomic tissue or organ assembly.

It has been long noticed that the mechanical signals are able to transfer into cells via an outside-in signaling pathway, which is mainly composed of ECM-integrin-FACs [38]. Our results further supported that the collagen I-β1 integrin-vinculin complex is most important molecules in mechanosensing of keratinocyte migration (Figs. 3, 4, 5 and 6). Here an important issue is why and how the static stretch, rather than the cyclic stretch, affects the cell migration. A cell is able to sense the deformation of elastic membrane initially and to reach the steady phase under a static stretch [39]. This interprets well why the early phase up-regulation was observed for the expressions of β1 integrin and vinculin within the first hour of stretch onset (Fig. 3a, b).

Downstream mechanotransduction of mechanical signals is also crucial to induce the asymmetric migration of HaCaT cells. As one of the major protein kinases, PI3K play a crucial role in mediating the predominant outward migration at least in a rapid phase (Fig. 5), suggesting that PI3K activity is required for keratinocyte migration [40]. ERK1/2 phosphorylation has also been known as a mechanotransductive indicator for integrin-based cell attachments [41]. The enhancement of phosphorylated-ERK1/2 found in the current work supported that ERK1/2 is sensitive to mechanical stimuli that mediates keratinocyte migration via β1 integrin (Fig. 6). Other signaling molecule, such as Rho GTPase Cdc42, is also contributed to the remodeling of actin cytoskeleton [42, 43]. High expression of Cdc42 is related to the formation of protrusive structures of the plasma membrane and formation of lamellipodia and filopodia [28]. It was also indicated that all the molecules of PI3K, ERK1/2, and Cdc42 are also dynamically activated in an early phase responses followed by a descending phase to a steady state, which is consistent with the dynamics of mechanosensing.

Migration and directed motility of keratinocytes on extracellular matrix components are known to go through β1 integrin-dependent PI3K and ERK pathways by stabilizing lamellipodia at the leading edge of cells in re-epithelializing wounds [44, 45]. Activation and localization of Cdc42 proteins on cell membrane are involved in the cell podia formation in keratinocytes, which may facilitate cell migration to accelerate wound healing [46]. Thus, keratinocyte migration requires spatiotemporal integration of signaling molecules that regulate cell body motility. Our results further supported that mechanical stretch, as an important stimulator, is able to induce remarkable expressions of Cdc42, PI3K and ERK1/2 at the leading edge within 1 h for C/S OUT case. These alterations were decreased or even disappeared when β1 integrins were knocked down (Figs. 4, 5 and 6). Meanwhile, cooperation of enhanced Cdc42 expression and pseudopodium formation for C/S OUT case with increased vinculin expression for C/S IN case enable HaCaT cells easier to adhere for C/S IN case and to migrate along C/S OUT direction. Importantly, cell movement is orchestrated by a variety of membrane-anchored and intracellular signaling proteins including β1 integrins, focal adhesion complex, Rho family of GTPases, PI3K and ERK1/2. These results provided, at least partially, the mechanotransductive mechanisms for asymmetric migration of HaCaT cells under mechanical stretch [16].

It was also noted in the current work that the presence of co-cultured HF cells are pre-requite for asymmetric migration of HaCaT cells, implying that the paracrine pathways of secreted growth factors by HF cells might be critical in regulating the migration dynamics [16, 47, 48]. While this topic is beyond the scope of the current work, it is worthwhile in the future work to understand the cross-talk of β1 integrin and epidermal growth factor interactions [49] or transforming growth factor [50, 51] via MAPK and PI3K signal pathway, which are crucial in regulating keratinocyte motility and migration during wound repair. Taken together, a simple pathway was proposed for mechanically-induced signal transduction pathways for asymmetric migration of keratinocytes in this work.

## Abbreviations

ECM: extracellular matrix
ERK1/2: extracellular signal-regulated kinase
FAC: focal adhesion complex
HF: human fibroblast
IF: immunofluorescence
MAPK: mitogenesis-associated protein kinase
PI3K: phosphatidylinositol 3-kinase
RNAi: RNA interference
RT-PCR: reverse transcription-polymerase chain reaction
siRNA: small interfering RNA
VAC: vacuum-assisted closure
WB: Western Blotting
C/N: co-culture and non-stretch
C/S: co-culture and stretch
IN: inward
OUT: outward
